# Judd–Ofelt Analysis and Emission Properties of Dy^3+^ Ions in Borogermanate Glasses

**DOI:** 10.3390/ma15249042

**Published:** 2022-12-17

**Authors:** Wojciech A. Pisarski

**Affiliations:** Institute of Chemistry, University of Silesia, Szkolna 9 Street, 40-007 Katowice, Poland; wojciech.pisarski@us.edu.pl

**Keywords:** borogermanate glasses, Dy^3+^ ions, absorption, emission, Judd–Ofelt analysis

## Abstract

Borogermanate glasses singly doped with Dy^3+^ ions were synthesized and then studied using the absorption and luminescence spectra. Spectroscopic changes of Dy^3+^ ions have been examined for compositional-dependent glasses with various molar ratios GeO_2_:B_2_O_3_. In this work, several spectroscopic parameters of Dy^3+^ ions were obtained experimentally and compared to the calculated values from the Judd–Ofelt theory. Luminescence spectra measured for borogermanate glasses consist of blue, yellow and red bands, which correspond to ^4^F_9/2_ → ^6^H_15/2_, ^4^F_9/2_ → ^6^H_13/2_ and ^4^F_9/2_ → ^6^H_11/2_ transitions of Dy^3+^, respectively. Luminescence lifetimes for the ^4^F_9/2_ excited state are reduced, whereas the stimulated emission cross-sections for the most intense ^4^F_9/2_ → ^6^H_13/2_ yellow transition of Dy^3+^ increase with increasing GeO_2_ and decreasing B_2_O_3_ concentrations in glass-hosts. Quantum efficiency of the ^4^F_9/2_ (Dy^3+^) excited state is nearly independent on molar ratios GeO_2_:B_2_O_3_. Attractive spectroscopic properties related to the ^4^F_9/2_ → ^6^H_13/2_ transition of Dy^3+^ ions are found for borogermanate glasses implying their potential utility for yellow laser action and solid-state lighting technology.

## 1. Introduction

Judd [1] and Ofelt [2] published their pioneering scientific works concerning the absorption intensities of rare earths 60 years ago. Based on the Judd–Ofelt (J-O) framework, several spectroscopic parameters for the optically active ions from the lanthanide series can be determined, which are really important from the optical and laser points of view. The most important of which are the radiative transition probability, the luminescence branching ratio and the radiative lifetime for the upper laser state of rare earth ions. The radiative transition probability and spectral linewidth for luminescent transition of rare earth ions can be used to calculate the peak stimulated emission cross-section, whereas measured emission lifetime and radiative lifetime calculated from the J-O framework are usually applied to estimate the quantum efficiency of the excited state. As a result, many glass matrices doped with rare earths can be quite well evaluated for a variety of possible applications such as for optical components and devices. Therefore, the J–O theory made a huge contribution to the development of optical and laser glasses in modern photonics. Since then, numerous published papers have been devoted to the study of glasses, glass-ceramics and other inorganic compounds singly or doubly doped with rare earth ions [3,4,5,6,7,8] using the theory on the intensities of 4f-4f electronic transitions introduced by Judd and Ofelt in 1962. In particular, the Judd–Ofelt analysis was performed for trivalent Nd^3+^ [9,10,11,12,13,14,15,16,17], Er^3+^ [18,19,20,21,22,23,24,25,26,27,28,29,30], Sm^3+^ [31,32,33,34,35], Pr^3+^ [36,37,38,39,40], Tm^3+^ [41,42,43,44], Ho^3+^ [45,46,47,48,49,50] and Dy^3+^ [51,52,53,54,55,56,57,58] ions in various inorganic glasses. The later trivalent rare earth ions, i.e., Dy^3+^ ions, were successfully used as an optical probe to study the luminescence behavior of inorganic glasses [59]. Recently, systematic investigations indicate that dysprosium doped glasses are excellent candidates for solid-state yellow lasers, white LEDs and other photonic device applications [60,61,62,63]. Their luminescence properties depend strongly on glass-network-modifiers [64], excitation wavelength and activator concentration [65].

In this work, the influence of glass-network-formers on the spectroscopic properties of dysprosium ions in borogermanate glasses has been examined in detail. Previous studies revealed that borogermanate glass belongs to amorphous systems with extremely different glass-network-formers B_2_O_3_ and GeO_2_, influencing the spectroscopic properties of Cr^3+^ and Eu^3+^ [66]. Further investigations revealed that borogermanate glasses are suitable to fabricate CsPbBr_3−x_I_x_ quantum dots with tunable visible emissions ranging from 577 nm to 672 nm [67]. In addition, they are able to accommodate rare earth ions. Thus, borogermanate glasses singly [68] and doubly [69] doped with rare earths are promising for luminescence applications. In recent years, borogermanate glass doped with Dy^3+^ has also been studied, but luminescence properties were analyzed as a function of activator content. Gökçe and Koçyiğit [70] suggest that Dy^3+^ doped gadolinium borogermanate glass matrix with the following composition 30B_2_O_3_-40GeO_2_-(30-x)Gd_2_O_3_-xDy_2_O_3_ (where x = 0.25, 0.5, 1) presents excellent luminescence properties and can be used for laser and white LED applications. Numerous works published in recent years are concerned with glasses and their spectroscopic properties varying with activator (Dy^3+^) content [71,72,73,74,75,76,77,78,79,80,81]. These aspects have been discussed along with the reported Dy^3+^ doped glass systems in an excellent paper published last year [82]. However, Dy^3+^-doped glasses have not been examined often as a function of chemical composition and the spectroscopic results are less documented in the literature. Previous studies for compositional-dependent germanosilicate glasses demonstrate that the intensities of emission bands are the largest for glass samples with the molar ratio GeO_2_:SiO_2_ = 3:1 and the optimal concentration of Dy^3+^ ions equal to 0.5 mol% [83]. It was also confirmed for zinc aluminoborosilicate glasses, where luminescence quenching is observed beyond 0.5 mol% Dy^3+^ ions suggesting the presence of an energy-transfer process through cross-relaxation channels [84].

This paper is concerned with Dy^3+^-doped borogermanate glasses and their emission properties varying with glass-host composition. Absorption and emission properties have been analyzed for glass samples, where the molar ratios of glass-network-formers GeO_2_:B_2_O_3_ are changed significantly and the activator concentration is equal to 0.5 mol% Dy^3+^. In particular, several spectroscopic parameters for Dy^3+^ ions were obtained experimentally and compared with theoretical values calculated from the J–O theory.

## 2. Materials and Methods

Borogermanate glasses doped with Dy^3+^ ions with the following composition given in molar%: (60-x)GeO_2_-xB_2_O_3_-30BaO-9.5Ga_2_O_3_-0.5Dy_2_O_3_ (x = 0, 5, 10, 20, 30, 40, 50, 60) were prepared previously and details are given in Ref. [85]. For better clarity, glass codes and chemical compositions of the studied samples varying with glass formers GeO_2_ and B_2_O_3_ marked in bold are shown in Table 1.

**Figure 1:**
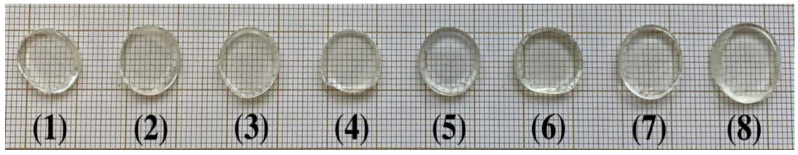
Photographs of the Dy^3+^ doped glass samples: GeO_2_-BaO-Ga_2_O_3_ (1), GeO_2_:B_2_O_3_ = 11:1 (2), GeO_2_:B_2_O_3_ = 5:1 (3), GeO_2_:B_2_O_3_ = 2:1 (4), GeO_2_:B_2_O_3_ = 1:1 (5), GeO_2_:B_2_O_3_ = 1:2 (6), GeO_2_:B_2_O_3_ = 1:5 (7) and B_2_O_3_-BaO-Ga_2_O_3_ (8).

Glass samples were synthesized using a melt-quenching technique. Starting components of high purity 99.99% (Aldrich Chemical Co., St. Louis, MO, USA) were used for glass synthesis. All oxide components were mixed in an agate mortar for homogenization. Then, glass batches were placed in an Al_2_O_3_ crucible and melted at temperature T = 1250 °C for t = 45 min in an electric furnace. The received glass samples were cooled to room temperature and then polished for optical measurements. Photographs of the Dy^3+^ doped glass samples are shown in Figure 1.

The refractive indices of glass series were determined using the Metricon 2010 prism coupler at a wavelength of 632.8 nm. The optical absorption spectra measurements were performed using the UV-VIS-NIR spectrophotometer (Cary 5000, Agilent Technology, Santa Clara, CA, USA). The emission spectra and decays were recorded using laser equipment, which consists of a Photon Technology International (PTI) Quanta-Master 40 (QM40) UV/VIS Steady State Spectrofluorometer (Photon Technology International, Birmingham, NJ, USA) with a xenon lamp as an excitation source, Nd:YAG laser (Opotek Opolette 355 LD, OPOTEK, Carlsband, CA, USA) with a tunable pulsed optical parametric oscillator, double 200 mm monochromator and multimode UVVIS PMT R928 detector (PTI Model 914). Resolution for emission spectra measurements was 0.1 nm, whereas decays were measured with an accuracy of 1 µs.

## 3. Theoretical Background

The measured oscillator strengths of transitions were obtained from the absorption bands of Dy^3+^ ions. They were estimated by measuring the areas under the absorption bands of Dy^3+^ ions using the equation:

(1) $$P_{\mathrm{meas}}=4.318\times 10^{-9}\int \varepsilon (\nu )d\nu$$

where: ∫ε(ν) represents the area under the absorption line and **ε**(**ν**) = A/(c × l), A indicates the absorbance, c is the concentration of the Dy^3+^ ion (in mol × l^−1^) and l denotes the optical path length. The theoretical oscillator strengths for each absorption transition of Dy^3+^ ions were calculated using the Judd–Ofelt theory [1,2]. The theoretical oscillator strength is defined as follows:

(2) $$P_{\mathrm{calc}}=\frac{8\pi^{2}mc{\left(n^{2}+2\right)}^{2}}{3h\lambda (2J+1)\cdot 9n}\times \sum_{t=2,4,6}\Omega_{t}{\left(<4f^{N}J\left\|U^{t}\right\|4f^{N}J^{\prime }>\right)}^{2}$$

where λ denotes the mean wavelength of each transition, whereas m, c, h and n are the mass of the electron, the velocity of light, the Planck constant and the refractive index of the medium, respectively. In this relation, ║U^t^║^2^ represents the square of the matrix elements of the unit tensor operator U^t^. The values of ║U^t^║^2^ used for Dy^3+^ were adopted from Ref. [86]. The theoretical oscillator strengths were compared to the experimental values obtained from the optical absorption spectra of Dy^3+^ ions in borogermanate glasses and the phenomenological intensity parameters Ω_t_ (where t = 2, 4, 6) were determined. The fit quality was expressed by the magnitude of the root-mean-square deviation. It was defined by rms = Σ (P_meas_ − P_calc_)^2^. These three Judd–Ofelt intensity parameters Ω_t_ (t = 2, 4, 6) were used to calculate the radiative transition probabilities, the luminescence branching ratios and the radiative lifetimes. The radiative transition probabilities A_J_ for the excited states of Dy^3+^ ions were calculated using the relation given below:

(3) $$A_{J}=\frac{64\pi^{4}e^{2}}{3h(2J+1)\lambda^{3}}\times \frac{n{\left(n^{2}+2\right)}^{2}}{9}\times \sum_{t=2,4,6}\Omega_{t}{\left(<4f^{N}J\left\|U^{t}\right\|4f^{N}J^{\prime }>\right)}^{2}$$

The luminescence branching ratio β is related to the relative intensities of transitions from the excited state to all terminal states of Dy^3+^ ions.

(4) $$\beta =\frac{A_{J}}{\sum_{i}A_{Ji}}$$

The radiative lifetime τ_rad_ is the inverse of the total radiative transition probability (the sum of the A_J_ terms). Its value was compared to the experimental lifetime received from the luminescence decay curve. Both calculated and measured lifetimes were applied to determine the quantum efficiency of an excited state η. The appropriate relations are given below:

(5) $$\tau_{\mathrm{rad}}=\frac{1}{\sum_{i}A_{Ji}}=\frac{1}{A_{T}}$$

(6) $$\eta =\frac{\tau_{m}}{\tau_{\mathrm{rad}}}\times 100\%$$

Finally, the emission linewidth Δλ referred as full width at half maximum (FWHM) and the radiative transition probability A_J_ were successfully used to calculate the peak stimulated emission cross-section σ_em_ using the following expression:

(7) $$\sigma_{\mathrm{em}}=\frac{\lambda_{p}^{4}}{8\pi cn^{2}\Delta \lambda }A_{J}$$

where λ_p_ is the peak emission wavelength for the electronic transition of Dy^3+^.

All theoretical and experimental spectroscopic parameters for Dy^3+^ ions in the studied borogermanate glasses are summarized in Table 2.

## 4. Results and Discussion

**Figure 2:**
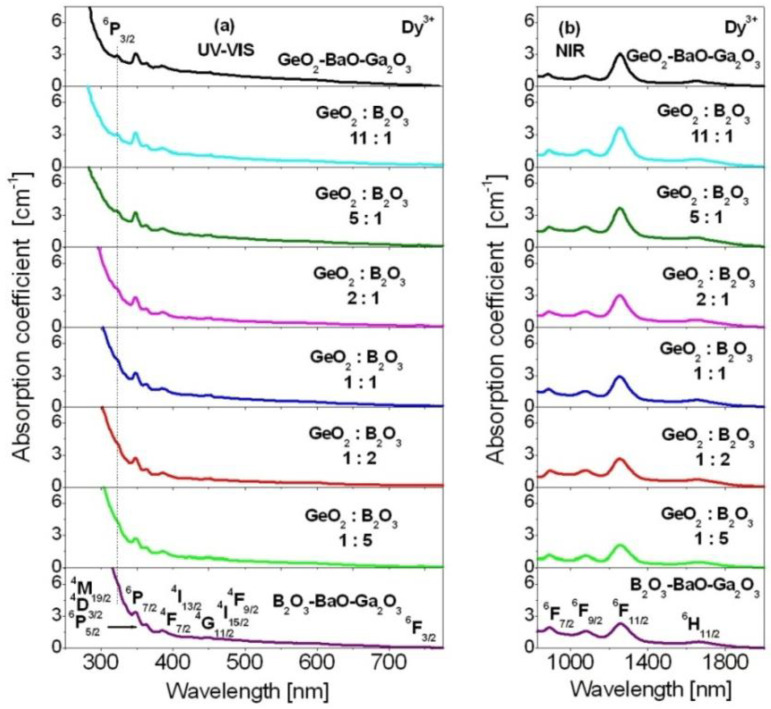
Absorption spectra for Dy^3+^ ions in borogermanate glasses with various molar ratios GeO_2_:B_2_O_3_ compared to GeO_2_-BaO-Ga_2_O_3_ and B_2_O_3_-BaO-Ga_2_O_3_ glasses. The spectra were measured in the UV-visible (**a**) and near-infrared (**b**) spectral ranges.

Judd–Ofelt analysis of Dy^3+^ ions in mixed borogermanate glasses with various GeO_2_:B_2_O_3_ molar ratios equal to 11:1, 5:1, 2:1, 1:1, 1:2 and 1:5 was carried out. Theoretical and experimental results were compared to GeO_2_-BaO-Ga_2_O_3_ and B_2_O_3_-BaO-Ga_2_O_3_ glasses. Absorption and emission properties have been examined for glass samples, where the concentration of Dy^3+^ ions was the same (0.5 mol%). Firstly, the absorption spectra measurements for Dy^3+^ ions in borogermanate glasses were carried out at room temperature. The absorption spectra of borogermanate glasses doped with Dy^3+^ ions were measured in the UV-visible and near-infrared spectral ranges, respectively. The spectra consist of inhomogeneously broadened absorption bands characteristic for 4f^9^-4f^9^ electronic transitions of Dy^3+^. The absorption bands correspond to transitions originating from the ^6^H_15/2_ ground state to the following excited states: ^6^H_11_*_/_*_2_, ^6^F_11_*_/_*_2_, ^6^F_9_*_/_*_2_, ^6^F_7_*_/_*_2_, ^6^F_3_*_/_*_2_, ^4^F_9_*_/_*_2_, ^4^I_15_*_/_*_2_, ^4^G_11_*_/_*_2_, ^4^I_13_*_/_*_2_, ^4^F_7_*_/_*_2_, (^4^M_19/2_+^4^D_3/2_+^6^P_5/2_), ^6^P_7/2_ and ^6^P_3/2_. The later transition, i.e., ^6^H_15/2_ → ^6^P_3/2_ transition, is clearly visible for GeO_2_-BaO-Ga_2_O_3_ glass contrary to B_2_O_3_-BaO-Ga_2_O_3_ glass. For mixed borogermanate glasses, the ^6^H_15/2_ → ^6^P_3/2_ transition of Dy^3+^ lies on the absorption edge. This indicates that the absorption edge is shifted to longer wavelengths from GeO_2_-BaO-Ga_2_O_3_ glass via mixed B_2_O_3_-GeO_2_-BaO-Ga_2_O_3_ compositions to B_2_O_3_-BaO-Ga_2_O_3_ glass, respectively. The absorption spectra are presented in Figure 2.

From the optical absorption spectra, the experimental oscillator strengths for Dy^3+^ ions have been determined. Owing to the standard procedure, the x-axes of absorption spectra were converted to wavenumbers (given in cm^−1^). In the next step, the baseline was fitted individually to each absorption band. The integrated areas of absorption bands were calculated. The intensities of absorption lines of Dy^3+^ ions presented in Figure 2 were estimated by measuring the areas under the bands, and then applied to determine the experimental oscillator strengths using relation (1). The commercially available software OriginPro was used during the calculation procedure.

**Figure 3:**
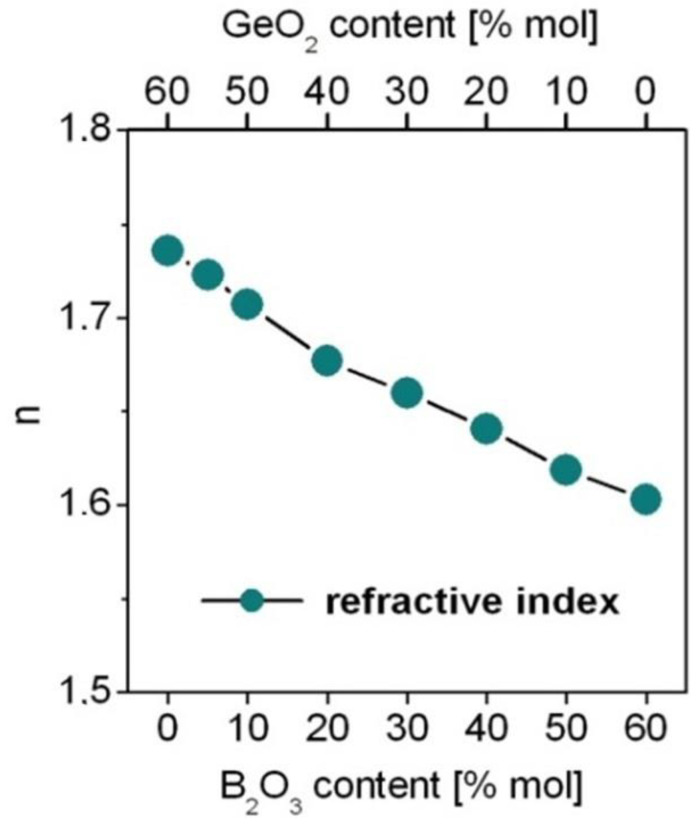
The refractive indices for borogermanate glasses.

The theoretical oscillator strengths for each transition of Dy^3+^ ions were calculated from the J–O framework (Part 3) using relation (2). In order to perform the analysis, the refractive index of the medium was used for calculations. The refractive index is changed from 1.736 for GeO_2_-BaO-Ga_2_O_3_ glass to 1.605 for B_2_O_3_-BaO-Ga_2_O_3_ glass. The refractive indices for the studied glass samples are schematized in Figure 3.

The experimental oscillator strengths from the absorption spectra and theoretical oscillator strengths were compared. They are shown in Table 3 and Table 4.

The main calculation process is related to three phenomenological Judd–Ofelt intensity parameters Ω_t_ (t = 2, 4, 6), which were obtained by comparison of the experimental oscillator strengths from the absorption spectra with the theoretical oscillator strengths from Equation (2) of the Judd–Ofelt framework (Part 3) using the fitting procedure. The quality of the fit shown in Table 3 and Table 4 expressed by the rms deviation defined by Σ(P_meas_ − P_calc_)^2^ (see Part 3) is quite good. The rms deviations for the studied glass systems varying with GeO_2_/B_2_O_3_ molar ratios are in the range 0.23–0.58 (×10^−6^). The error is within the acceptable range compared to similar glass doped with Dy^3+^ [70], which was studied using the Judd–Ofelt framework. The three Judd–Ofelt intensity parameters Ω_t_ (t = 2, 4, 6) are necessary to calculate some spectroscopic parameters such as the radiative transition probabilities and the luminescence branching ratios, and then the radiative lifetimes, the quantum efficiencies of excited states and the peak stimulated emission cross-sections for electronic transitions of Dy^3+^ ions. The three Judd–Ofelt intensity parameters Ω_t_ (t = 2, 4, 6) for Dy^3+^ ions in borogermanate glasses are given in Table 5.

It is generally accepted that the phenomenological Judd–Ofelt intensity parameter Ω_2_ reflects the asymmetry of the environment of trivalent dysprosium ions. In other words, the values of Ω_2_ exhibit the degree of covalency between Dy^3+^ ions and their nearest surroundings. For the studied glass systems, the Judd–Ofelt parameter Ω_2_ is reduced from 8.73 × 10^−20^ cm^2^ for GeO_2_-BaO-Ga_2_O_3_ glass to 5.92 × 10^−20^ cm^2^ for B_2_O_3_-BaO-Ga_2_O_3_ glass suggesting more ionic bonding between Dy^3+^ ions and ligands with increasing B_2_O_3_ concentration. The results are in good agreement with values of Ω_2_ calculated for similar germanate or germanate-tellurite glasses based on Na_2_O-MgO-Al_2_O_3_-GeO_2_ composition (Ω_2_ = 8.62 × 10^−20^ cm^2^) referred to as NMAG [87] and Na_2_O-ZnO-PbO-GeO_2_-TeO_2_ composition (Ω_2_ = 7.34 × 10^−20^ cm^2^) referred to as NZPGT [88] as well as obtained for similar borate glasses based on the B_2_O_3_-CaF_2_-CaO-BaO-Al_2_O_3_ system (Ω_2_ = 5.98 × 10^−20^ cm^2^) referred to as CFB [89] and B_2_O_3_-ZnO-Al_2_O_3_-Bi_2_O_3_ (Ω_2_ = 6.20 × 10^−20^ cm^2^) referred to as ZnAlBiB [90]. Following that, the Judd–Ofelt intensity parameters Ω_4_ and Ω_6_ are structure-dependent, i.e., the parameter Ω_4_ describes the viscosity of the glass medium while the parameter Ω_6_ is connected with the rigidity of the glass medium. Interestingly, GeO_2_-BaO-Ga_2_O_3_ glass and borogermanate glasses with lower B_2_O_3_ content (GeO_2_:B_2_O_3_ from 11:1 to 2:1) exhibit Ω_4_ > Ω_6_, whereas B_2_O_3_-BaO-Ga_2_O_3_ glass and borogermanate glasses with relatively higher B_2_O_3_ content (GeO_2_:B_2_O_3_ = 1:2 and 1:5) possess Ω_4_ < Ω_6_ (Table 3). The same situation was observed earlier for germanate, germanate-tellurite and tellurite glasses [87,87,91], where Ω_4_ > Ω_6_ contrary to borate or phosphate glasses [90,92,93], where Ω_4_ < Ω_6_. However, further investigations for borate-based glasses suggest that Ω_4_ < Ω_6_ can be changed to Ω_4_ > Ω_6_ with decreasing Dy^3+^ concentration [94]. For glass with GeO_2_:B_2_O_3_ = 1:1 both the Judd–Ofelt parameters Ω_4_ and Ω_6_ are nearly the same as Dy^3+^ doped silicate glass based on SiO_2_–Al_2_O_3_–PbF_2_–AlF_3_–YbF_3_–DyF_3_ composition [95].

It was concluded that the intensity parameters Ω_4_ and Ω_6_ depend not only on the viscosity and rigidity, but they are also affected by the acidity and basicity of the glass-host, i.e., the highest Ω_4_ and Ω_6_ indicates the lowest basicity of the glass and the highest hardness [83]. In particular, the parameter Ω_6_ is reduced systematically with increasing basicity and decreasing rigidity of the glass [96]. The calculation results given in Table 5 clearly indicate that the intensity parameter Ω_6_ increases from GeO_2_-BaO-Ga_2_O_3_ glass to B_2_O_3_-BaO-Ga_2_O_3_ glass. The values of Ω_6_ are larger for glass samples containing higher B_2_O_3_ concentrations suggesting their lower basicity and higher rigidity. Further studies suggest that the Judd–Ofelt intensity parameters Ω_4_ and Ω_6_ not only influence the physicochemical properties of glasses but also strongly affect the radiative transition probabilities as a result of the interaction between trivalent dysprosium ions and their nearest environments [97].

Following that, the spectroscopic quality parameter χ referred to as the magnitude of Ω_4_/Ω_6_ belongs to important factors characterizing the optical potential of the currently prepared glass. It was presented and discussed in detail for several glass systems doped with Dy^3+^ ions [91]. For borogermanate glass systems, the values of χ are relatively large, which demonstrates quite well the intense luminescent transitions of Dy^3+^ ions. Luminescence studies for multicomponent glass based on B_2_O_3_–Bi_2_O_3_–SrO–Al_2_O_3_–PbO–Dy_2_O_3_ revealed that the spectroscopic quality factor χ ≥ 0.50, can be suggested as a good optical candidate for the lasing action of dysprosium ions [80].

The three phenomenological J–O intensity parameters Ω_t_ (t = 2, 4, 6) were applied to calculate the radiative transition probabilities and the luminescence branching ratios using the appropriate Relations (3) and (4) given in Part 3. The results are summarized in Table 6 and Table 7. The total radiative transition probability A_TOTAL_ referred to as the sum of the A_J_ terms from the ^4^F_9/2_ excited state of dysprosium ions increases with increasing GeO_2_ concentration. The value of A_TOTAL_ changed from 904 s^−1^ for B_2_O_3_-BaO-Ga_2_O_3_ glass to 933 s^−1^ (GeO_2_:B_2_O_3_ = 1:5), 1065 s^−1^ (GeO_2_:B_2_O_3_ = 1:2), 1095 s^−1^ (GeO_2_:B_2_O_3_ = 1:1), 1124 s^−1^ (GeO_2_:B_2_O_3_ = 2:1), 1278 s^−1^ (GeO_2_:B_2_O_3_ = 5:1), 1311 s^−1^ (GeO_2_:B_2_O_3_ = 11:1) and 1495 s^−1^ for GeO_2_-BaO-Ga_2_O_3_ glass, respectively. In all cases, the luminescence branching ratio is the highest for the ^4^F_9/2_ → ^6^H_13/2_ electronic transition of Dy^3+^ ions at 573 nm. Its value changed from 69.1% to 74.5% depending on the chemical composition of the glass-host. The calculation results suggest that the studied borogermanate glass systems are promising for yellow emission independently on molar ratios GeO_2_:B_2_O_3_. The luminescence spectra measurements confirm this hypothesis.

**Figure 4:**
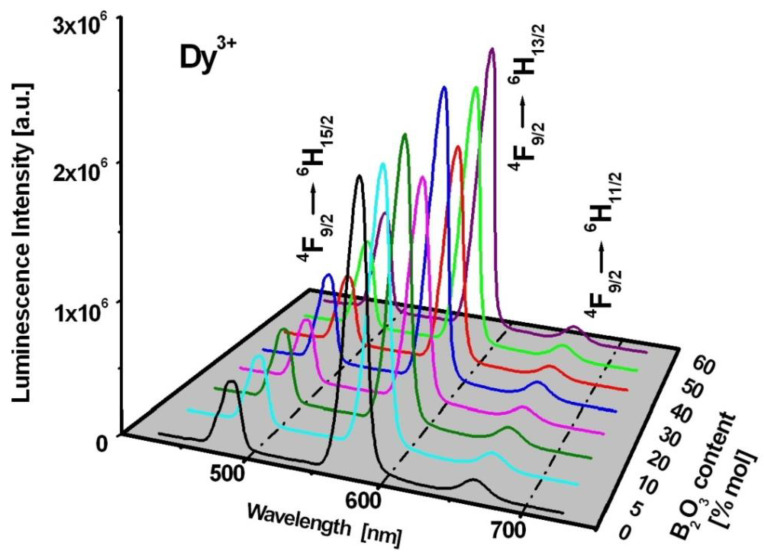
Luminescence spectra for borogermanate glasses doped with Dy^3+^.

**Figure 5:**
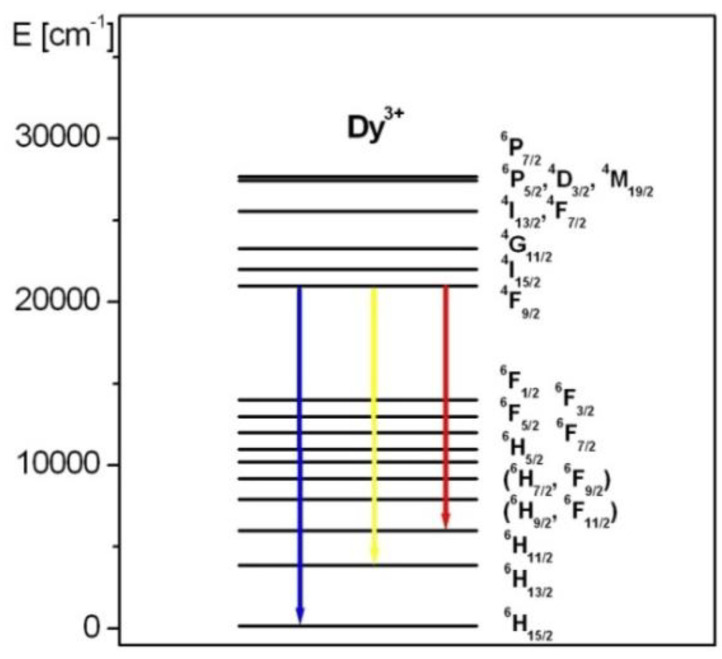
Energy level diagram for Dy^3+^ ions. Luminescent transitions are also indicated.

Figure 4 presents the luminescence spectra of Dy^3+^ ions in borogermanate glasses. The spectra for glasses based on B_2_O_3_-BaO-Ga_2_O_3_ and GeO_2_-BaO-Ga_2_O_3_ are also indicated. The emission spectra show three characteristic bands of Dy^3+^ ions located at blue, yellow and red spectral range. These luminescence bands are attributed to ^4^F_9/2_ → ^6^H_15/2_ (blue), ^4^F_9/2_ → ^6^H_13/2_ (yellow) and ^4^F_9/2_ → ^6^H_11/2_ transitions of trivalent dysprosium. In previous work [85], the influence of glass former (GeO_2_), oxide (CaO/SrO/BaO) and fluoride (CaF_2_/SrF_2_/BaF_2_) glass modifiers on spectral properties, the yellow-to-blue luminescence intensity ratios and CIE coordinates of Dy^3+^ in borate-based glasses have been examined in detail. The studies revealed that the CIE chromaticity coordinates (x, y) are changed significantly with molar ratios GeO_2_:B_2_O_3_ in glass composition. The CIE coordinates are changed from (x = 0.405, y = 0.452) to (x = 0.430, y = 0.472) with increasing GeO_2_ content, which contributes to color modification of the borogermanate glass system from greenish to yellowish. These experimental results are presented and discussed in the previously published work [85]. The luminescent transitions of Dy^3+^ ions are indicated in the energy level diagram shown in Figure 5.

**Figure 6:**
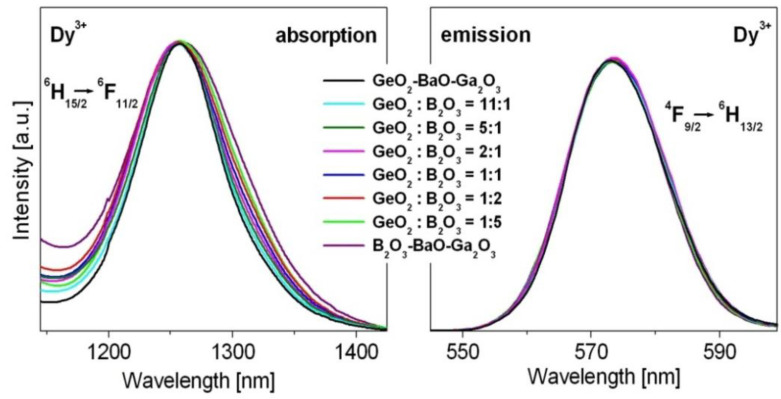
Hypersensitive absorption and emission transitions Dy^3+^ ions.

The luminescent results presented in Figure 4 indicate that the intensities are the highest for yellow bands related to the ^4^F_9/2_ → ^6^H_13/2_ transition of Dy^3+^, independently on GeO_2_:B_2_O_3_ ratios. In addition, the ^4^F_9/2_ → ^6^H_13/2_ transition of Dy^3+^ ions is so-called hypersensitive transition, which follows the selection rules ⏐S⏐ = 0, ⏐ΔL⏐ ≤ 2 and ⏐ΔJ⏐ ≤ 2. The emission intensities as well as the spectral profiles and positions are very sensitive to even small changes of the nearest environment around dysprosium ions. The same situation is observed for the absorption band centered near 1250 nm due to transition originating from the ^6^H_15/2_ ground state to the ^6^F_11/2_ state. Figure 6 shows hypersensitive absorption and emission transitions of Dy^3+^ varying with GeO_2_:B_2_O_3_ molar ratios. In order to compare the spectral profile and position of hypersensitive transitions, the spectra were normalized. Spectroscopic analysis indicates that the spectra are broader with increasing B_2_O_3_ content. These effects are significantly stronger for absorption than emission bands.

**Figure 7:**
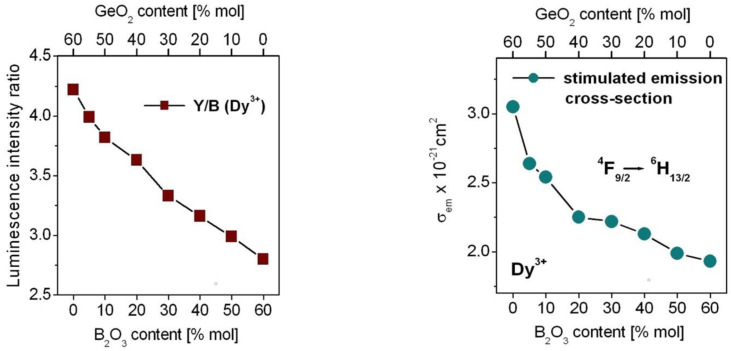
Luminescence intensity ratio Y/B (on **left**) and the peak stimulated emission cross-section for the ^4^F_9/2_ → ^6^H_13/2_ transition of Dy^3+^ ions in borogermanate glasses (on **right**).

Further luminescent studies suggest that yellow-to-blue factor Y/B (Dy^3+^) due to the ratio of the integrated emission intensities (^4^F_9/2_ → ^6^H_13/2_)/(^4^F_9/2_ → ^6^H_15/2_) is changed significantly with molar ratios GeO_2_:B_2_O_3_ in glass composition. The values of Y/B (Dy^3+^) are reduced from 4.22 for GeO_2_-BaO-Ga_2_O_3_ glass to 2.80 for B_2_O_3_-BaO-Ga_2_O_3_ glass with increasing B_2_O_3_ concentration suggesting more ionic bonding between Dy^3+^ ions and surrounding ligands. The results are in a good agreement with the calculated values of the intensity parameters Ω_2_, which decrease from 8.73 for GeO_2_-BaO-Ga_2_O_3_ glass to 5.92 for B_2_O_3_-BaO-Ga_2_O_3_ glass indicating more ionic bonding in character. It was schematized on Figure 7.

The same situation is also observed for the peak stimulated emission cross-section calculated from Equation (7) in Part 3 for the ^4^F_9/2_ → ^6^H_13/2_ transition of Dy^3+^ at 573 nm, which is decreased with increasing B_2_O_3_ content (Figure 7). The values of *σ*_em_ given in 10^−21^ cm^2^ are changed from 3.05 for GeO_2_-BaO-Ga_2_O_3_ glass to 2.63 (GeO_2_:B_2_O_3_ = 11:1), 2.54 (GeO_2_:B_2_O_3_ = 5:1), 2.25 (GeO_2_:B_2_O_3_ = 2:1), 2.22 (GeO_2_:B_2_O_3_ = 1:1), 2.13 (GeO_2_:B_2_O_3_ = 1:2), 1.99 (GeO_2_:B_2_O_3_ = 1:5) and 1.93 for B_2_O_3_-BaO-Ga_2_O_3_ glass, respectively.

From the series of the studied glass samples, the stimulated emission cross section is the highest (*σ*_em_ = 3.05 *×* 10^−21^ cm^2^) for GeO_2_-BaO-Ga_2_O_3_ glass. Its value is comparable to the one obtained for the ^4^F_9/2_ → ^6^H_13/2_ transition of Dy^3+^ ions in germanate-tellurite glasses based on GeO_2_-TeO_2_-SrF_2_ composition (*σ*_em_ = 3.1 *×* 10^−21^ cm^2^) referred as GTS [98] and Na_2_O-ZnO-PbO-GeO_2_-TeO_2_ composition (*σ*_em_ = 3.66 *×* 10^−21^ cm^2^) known as NZPGT [88].

**Figure 8:**
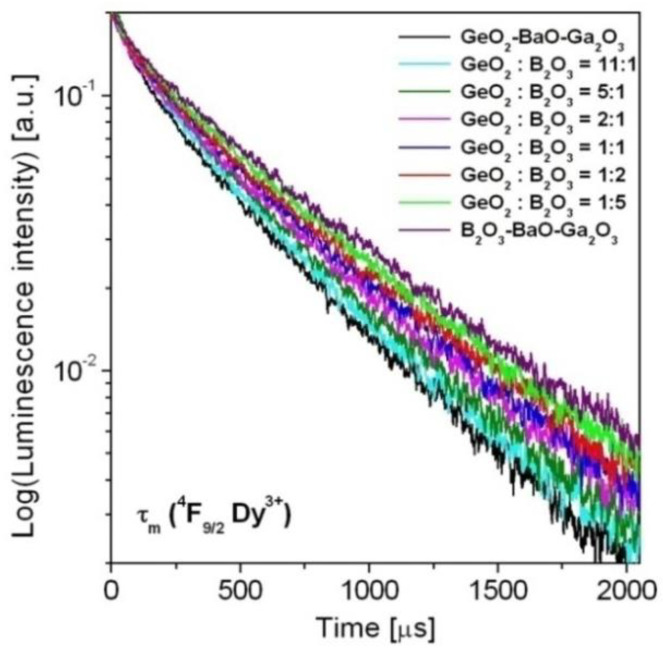
Luminescence decay curves for the ^4^F_9/2_ state of Dy^3+^ ions in borogermanate glasses.

Finally, luminescence decays from the ^4^F_9/2_ state of Dy^3+^ ions have been analyzed in detail. Decay curves for the ^4^F_9/2_ (Dy^3+^) state in borogermanate glasses were measured under excitation 454 nm and monitoring emission wavelength 573 nm. The luminescence decay curves for Dy^3+^ are presented in Figure 8. The obtained results clearly demonstrated that decays are longer with increasing B_2_O_3_ concentration in glass composition.

**Figure 9:**
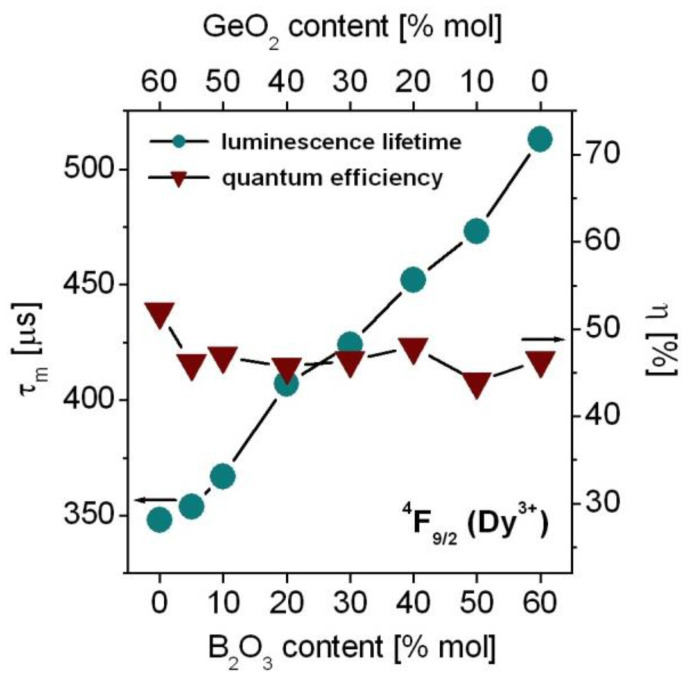
Luminescence lifetimes and quantum efficiencies for the ^4^F_9/2_ state of Dy^3+^ ions in borogermanate glasses.

Based on decay curve measurements, luminescence lifetimes for the ^4^F_9/2_ state of Dy^3+^ ions were determined. Next, measured lifetimes were compared to the radiative lifetimes (Equation (5), Part 3) calculated from the J–O theory. Both measured *τ*_m_ and radiative *τ*_rad_ lifetimes were used to calculate quantum efficiency (Equation (6), Part 3). The measured lifetimes and quantum efficiencies for the ^4^F_9/2_ state of Dy^3+^ varying with GeO_2_:B_2_O_3_ molar ratios are schematically shown in Figure 9.

The ^4^F_9/2_ lifetime of Dy^3+^ ions in GeO_2_-BaO-Ga_2_O_3_ glass is close to 348 µs and its value is comparable to the results (*τ*_m_ = 356 µs) obtained for lead germanate glass based on PbO-Ga_2_O_3_-GeO_2_ [99]. The experimental values of *τ*_m_ for mixed borogermanate glasses are equal to 352 µs (GeO_2_:B_2_O_3_ = 11:1), 367 µs (GeO_2_:B_2_O_3_ = 5:1), 407 µs (GeO_2_:B_2_O_3_ = 2:1), 424 µs (GeO_2_:B_2_O_3_ = 1:1), 452 µs (GeO_2_:B_2_O_3_ = 1:2), and 473 µs (GeO_2_:B_2_O_3_ = 1:5). The ^4^F_9/2_ lifetime of Dy^3+^ ions is the highest (*τ*_m_ = 513 µs) for B_2_O_3_-BaO-Ga_2_O_3_ glass. In contrast to the dependences of luminescence intensity ratio Y/B and the peak stimulated emission cross-section for the ^4^F_9/2_^®^ ^6^H_13/2_ transition (Figure 7), the luminescence lifetime for the ^4^F_9/2_ state of Dy^3+^ increases with increasing B_2_O_3_ content. It is experimentally evidenced that the non-radiative multiphonon relaxation probabilities of rare earth ions are increased significantly with increasing phonon energy from GeO_2_ to B_2_O_3_. Glass based on GeO_2_-BaO-Ga_2_O_3_ (~800 cm^−1^) has relatively smaller phonon energy than B_2_O_3_-BaO-Ga_2_O_3_ glass (~1400 cm^−1^). Thus, the measured lifetimes of rare earth ions are reduced from GeO_2_ to B_2_O_3_ because multiphonon relaxation probabilities become higher with increasing B_2_O_3_ content. For example, this situation is observed for Er^3+^ ions, where the energy separation between the excited state ^4^I_13/2_ and next lower-lying ground state ^4^I_15/2_ is relatively small and non-radiative multiphonon relaxation provides an important contribution to the total relaxation process. The opposite effects are observed for other rare earth ions such as Tb^3+^, Eu^3+^ or Dy^3+^, where the energy gaps between the interacting levels are relatively large and non-radiative relaxation probabilities are negligibly small. Thus, luminescence lifetimes ^4^F_9/2_ (Dy^3+^) are nearly equal to radiative lifetimes calculated from the J–O theory and their experimental values increase from GeO_2_-BaO-Ga_2_O_3_ glass to B_2_O_3_-BaO-Ga_2_O_3_ glass. It was also confirmed earlier by the measurements of luminescence decay curves for rare earth ions in heavy metal oxide glasses referred to as HMOG. Previously published work clearly demonstrated that the dependence of experimental luminescence lifetimes on the phonon energies of HMOG glass systems is completely different for the ^5^D_0_ state of Eu^3+^ than for the ^4^I_13/2_ state of Er^3+^ [100].

Further studies indicate that the quantum efficiency of excited state ^4^F_9/2_ (Dy^3+^) is almost unchanged with increasing B_2_O_3_ concentration. The quantum efficiency for the ^4^F_9/2_ state of Dy^3+^ in mixed borogermanate glasses seems to be 47 ± 1%, independently of GeO_2_:B_2_O_3_ molar ratios. For GeO_2_-BaO-Ga_2_O_3_ glass (*η* = 52%) the quantum efficiency is above 50%. The results obtained for borogermanate glasses singly doped with Dy^3+^ ions suggest their potential luminescent applications in the yellow spectral range [71,101].

## 5. Conclusions

Borogermanate glasses doped with Dy^3+^ have been studied experimentally and theoretically using the Judd–Ofelt framework. Based on absorption and emission spectra measurements, several spectroscopic parameters for Dy^3+^ ions were determined, such as the measured and calculated oscillator strengths, the Judd–Ofelt intensity parameters, the radiative transition probabilities, the luminescence branching ratios, the peak stimulated emission cross-sections, the measured and radiative (calculated) luminescence lifetimes and the quantum efficiencies of excited state. They have been examined as a function of GeO_2_:B_2_O_3_ molar ratios in glass composition. The systematic investigations demonstrated that the peak stimulated emission cross-sections for the most intense ^4^F_9/2_ → ^6^H_13/2_ yellow transition of Dy^3+^ ions decrease, whereas the ^4^F_9/2_ luminescence lifetimes are enhanced with increasing B_2_O_3_ concentration. The quantum efficiencies for the ^4^F_9/2_ state of Dy^3+^ ions are close to η = 47 ± 1% and their values are nearly independent of GeO_2_:B_2_O_3_ ratios. It was suggested that the results for borogermanate glasses doped with Dy^3+^ are attractive for yellow luminescence, providing an important contribution to the development of optical glasses and celebrating the 60th anniversary of the Judd–Ofelt theory.

## Figures and Tables

**Table 1:** Glass codes and chemical compositions for glass samples doped with Dy^3+^ ions.

No	Glass Code	Chemical Composition [mol%]
(1)	GeO_2_-BaO-Ga_2_O_3_	**60GeO_2_**-30BaO-9.5Ga_2_O_3_-0.5Dy_2_O_3_
(2)	GeO_2_:B_2_O_3_ = 11:1	**55GeO_2_-5B_2_O_3_**-30BaO-9.5Ga_2_O_3_-0.5Dy_2_O_3_
(3)	GeO_2_:B_2_O_3_ = 5:1	**50GeO_2_-10B_2_O_3_**-30BaO-9.5Ga_2_O_3_-0.5Dy_2_O_3_
(4)	GeO_2_:B_2_O_3_ = 2:1	**40GeO_2_-20B_2_O_3_**-30BaO-9.5Ga_2_O_3_-0.5Dy_2_O_3_
(5)	GeO_2_:B_2_O_3_ = 1:1	**30GeO_2_-30B_2_O_3_**-30BaO-9.5Ga_2_O_3_-0.5Dy_2_O_3_
(6)	GeO_2_:B_2_O_3_ = 1:2	**20GeO_2_-40B_2_O_3_**-30BaO-9.5Ga_2_O_3_-0.5Dy_2_O_3_
(7)	GeO_2_:B_2_O_3_ = 1:5	**10GeO_2_-50B_2_O_3_**-30BaO-9.5Ga_2_O_3_-0.5Dy_2_O_3_
(8)	B_2_O_3_-BaO-Ga_2_O_3_	**60B_2_O_3_**-30BaO-9.5Ga_2_O_3_-0.5Dy_2_O_3_

**Table 2:** Theoretical and experimental spectroscopic parameters for Dy^3+^ in borogermanate glasses.

Parameters	Symbols	Units
Theoretical oscillator strength	P_calc_	-
Measure oscillator strength	P_meas_	-
Judd–Ofelt intensity parameters	Ω_t_ (t = 2, 4, 6)	10^−20^ cm^2^
Spectroscopic quality parameter	χ (Ω_4_/Ω_6_)	-
Radiative transition probability	A_J_	s^−1^
Total radiative transition probability	A_T_	s^−1^
Luminescence branching ratio	β	%
Radiative lifetime	τ_rad_	µs
Measured lifetime	τ_meas_	µs
Quantum efficiency	η	%
Peak emission wavelength	λ_p_	nm
Emission linewidth	Δλ	nm
Full width at half maximum	FWHM	nm
Peak stimulated emission cross-section	σ_em_	10^−21^ cm^2^

**Table 3:** Measured and calculated oscillator strengths (P × 10^−6^) for Dy^3+^ ions in GeO_2_-BaO-Ga_2_O_3_ glass and mixed borogermanate glasses with GeO_2_:B_2_O_3_ = 11:1, 5:1 and 2:1.

Levels	Energy [cm^−1^]	GeO_2_-BaO-Ga_2_O_3_		GeO_2_:B_2_O_3_ = 11:1		GeO_2_:B_2_O_3_ = 5:1		GeO_2_:B_2_O_3_ = 2:1	
		P_meas_	P_calc_	P_meas_	P_calc_	P_meas_	P_calc_	P_meas_	P_calc_
^6^H_11/2_ ^6^F_11/2_ ^6^F_9/2_ ^6^F_7/2_ ^6^F_3/2_ ^4^F_9/2_ ^4^I_15/2_ ^4^G_11/2_ ^4^F_7/2_,^4^I_13/2_ ^4^M_19/2_,^4^D_3/2_,^6^P_5/2_ ^6^P_7/2_	6040 7960 9300 11,290 13,450 21,200 22,200 23,600 25,900 27,500 28,700	1.260 8.220 1.580 1.410 0.090 0.150 0.330 0.075 0.820 0.730 3.070	1.109 8.240 1.783 1.391 0.117 0.105 0.360 0.063 0.601 0.932 2.364	1.060 8.150 1.500 1.260 0.090 0.080 0.330 0.070 0.750 0.650 3.200	0.980 8.161 1.677 1.168 0.092 0.087 0.311 0.069 0.597 0.754 2.648	1.190 7.600 1.500 1.580 0.100 0.080 0.310 0.065 0.750 0.720 2.910	1.089 7.613 1.748 1.417 0.122 0.107 0.357 0.057 0.573 0.960 2.157	1.120 6.980 1.470 1.310 0.100 0.070 0.350 0.090 0.900 0.700 2.770	0.987 6.998 1.651 1.298 0.110 0.098 0.322 0.057 0.546 0.871 2.160

**Table 4:** Measured and calculated oscillator strengths (P × 10^−6^) for Dy^3+^ ions in B_2_O_3_-BaO-Ga_2_O_3_ glass and mixed borogermanate glasses with GeO_2_:B_2_O_3_ = 1:1, 1:2 and 1:5.

Levels	Energy [cm^−1^]	GeO_2_:B_2_O_3_ = 1:1		GeO_2_:B_2_O_3_ = 1:2		GeO_2_:B_2_O_3_ = 1:5		B_2_O_3_-BaO-Ga_2_O_3_	
		P_meas_	P_calc_	P_meas_	P_calc_	P_meas_	P_calc_	P_meas_	P_calc_
^6^H_11/2_ ^6^F_11/2_ ^6^F_9/2_ ^6^F_7/2_ ^6^F_3/2_ ^4^F_9/2_ ^4^I_15/2_ ^4^G_11/2_ ^4^F_7/2_,^4^I_13/2_ ^4^M_19/2_,^4^D_3/2_,^6^P_5/2_ ^6^P_7/2_	6040 7960 9300 11,290 13,450 21,200 22,200 23,600 25,900 27,500 28,700	1.160 6.850 1.650 1.140 0.140 0.130 0.350 0.075 0.750 0.770 2.470	1.000 6.871 1.690 1.346 0.115 0.102 0.328 0.056 0.551 0.906 2.160	1.170 6.120 1.720 1.860 0.100 0.090 0.360 0.070 0.800 0.870 2.120	1.137 6.125 1.867 1.699 0.155 0.130 0.386 0.046 0.549 1.186 1.745	1.120 5.600 1.580 1.400 0.100 0.080 0.340 0.065 0.750 0.880 1.970	1.004 5.615 1.659 1.481 0.134 0.113 0.339 0.043 0.496 1.029 1.637	1.090 5.390 1.740 1.650 0.160 0.100 0.330 0.085 0.810 0.740 2.000	1.038 5.397 1.810 1.612 0.146 0.123 0.353 0.046 0.531 1.117 1.800

**Table 5:** Judd–Ofelt intensity parameters for Dy^3+^ ions in the studied glass systems.

Glasses	Judd–Ofelt Intensity Parameters Ω_t_ (t = 2, 4, 6) [in 10^−20^ cm^2^ Units]			χ (Ω_4_/Ω_6_)
	Ω_2_	Ω_4_	Ω_6_	
GeO_2_-BaO-Ga_2_O_3_ GeO_2_:B_2_O_3_ = 11:1 GeO_2_:B_2_O_3_ = 5:1 GeO_2_:B_2_O_3_ = 2:1 GeO_2_:B_2_O_3_ = 1:1 GeO_2_:B_2_O_3_ = 1:2 GeO_2_:B_2_O_3_ = 1:5 B_2_O_3_-BaO-Ga_2_O_3_	8.73 ± 0.22 8.42 ± 0.17 8.09 ± 0.23 7.52 ± 0.21 7.45 ± 0.19 6.72 ± 0.17 6.27 ± 0.16 5.92 ± 0.13	1.44 ± 0.21 1.60 ± 0.15 1.52 ± 0.22 1.35 ± 0.20 1.37 ± 0.18 1.11 ± 0.15 1.06 ± 0.14 1.18 ± 0.12	1.33 ± 0.14 1.03 ± 0.10 1.40 ± 0.14 1.29 ± 0.13 1.36 ± 0.12 1.87 ± 0.10 1.65 ± 0.10 1.81 ± 0.08	1.08 1.55 1.09 1.05 1.00 0.60 0.64 0.65

**Table 6:** The radiative transition probabilities and luminescence branching ratios for Dy^3+^ ions in GeO_2_-BaO-Ga_2_O_3_ glass and mixed borogermanate glasses with GeO_2_:B_2_O_3_ = 11:1, 5:1 and 2:1.

Transition	λ [nm]	GeO_2_-BaO-Ga_2_O_3_		GeO_2_:B_2_O_3_ = 11:1		GeO_2_:B_2_O_3_ = 5:1		GeO_2_:B_2_O_3_ = 2:1	
		A_J_ [s^−1^]	β	A_J_ [s^−1^]	β	A_J_ [s^−1^]	β	A_J_ [s^−1^]	β
^4^F_9/2_ → ^6^F_1/2_ ^6^F_3/2_ ^6^F_5/2_ ^6^F_7/2_ ^6^H_5/2_ ^6^H_7/2_ ^6^F_9/2_ ^6^F_11/2_ ^6^H_9/2_ ^6^H_11/2_ ^6^H_13/2_ ^6^H_15/2_	1373 1275 1156 992 918 836 830 749 746 662 573 480	>0.1 >0.1 15 6 4 19 11 36 25 125 1088 165	- - 0.010 0.004 0.003 0.013 0.007 0.024 0.017 0.084 0.728 0.110	>0.1 >0.1 13 4 3 17 10 32 23 108 976 125	- - 0.010 0.003 0.002 0.013 0.008 0.024 0.018 0.082 0.745 0.095	>0.1 >0.1 12 4 3 16 9 30 22 100 932 150	- - 0.009 0.004 0.003 0.013 0.007 0.023 0.017 0.078 0.729 0.117	>0.1 >0.1 11 4 3 15 8 26 19 88 818 132	- - 0.010 0.004 0.003 0.013 0.007 0.023 0.017 0.078 0.728 0.117

**Table 7:** The radiative transition probabilities and luminescence branching ratios for Dy^3+^ ions in B_2_O_3_-BaO-Ga_2_O_3_ glass and mixed borogermanate glasses with GeO_2_:B_2_O_3_ = 1:1, 1:2 and 1:5.

Transition	λ [nm]	GeO_2_:B_2_O_3_ = 1:1		GeO_2_:B_2_O_3_ = 1:2		GeO_2_:B_2_O_3_ = 1:5		B_2_O_3_-BaO-Ga_2_O_3_	
		A_J_ [s^−1^]	β	A_J_ [s^−1^]	β	A_J_ [s^−1^]	β	A_J_ [s^−1^]	β
^4^F_9/2_ → ^6^F_1/2_ ^6^F_3/2_ ^6^F_5/2_ ^6^F_7/2_ ^6^H_5/2_ ^6^H_7/2_ ^6^F_9/2_ ^6^F_11/2_ ^6^H_9/2_ ^6^H_11/2_ ^6^H_13/2_ ^6^H_15/2_	1373 1275 1156 992 918 836 830 749 746 662 573 480	>0.1 >0.1 10 4 3 15 8 25 19 85 792 134	- - 0.009 0.004 0.003 0.014 0.007 0.023 0.017 0.078 0.723 0.122	>0.1 >0.1 9 4 3 15 7 23 18 76 743 167	- - 0.008 0.004 0.003 0.014 0.007 0.022 0.016 0.071 0.698 0.157	>0.1 >0.1 8 3 2 13 6 20 16 67 656 142	- - 0.009 0.003 0.002 0.014 0.007 0.021 0.017 0.072 0.703 0.152	>0.1 >0.1 7 3 2 13 6 19 15 63 625 151	- - 0.008 0.003 0.002 0.014 0.007 0.021 0.017 0.070 0.691 0.167

## Data Availability

The data presented in this study are available on request from the author.
